# Peripheral odontogenic keratocyst. A Case report

**DOI:** 10.4317/jced.60033

**Published:** 2023-02-01

**Authors:** María Hornillos-de Villota, Marta Mª Pampin-Martínez, Mª José Moran-Soto, José-Luis Cebrián-Carretero

**Affiliations:** 1 Departamento de Cirugía Oral y Maxilofacial, Hospital Universitario La Paz. Madrid, Spain

## Abstract

Odontogenic keratocysts (OKCs) are benign cysts arising from the dental lamina and its remnants. They are most commonly located in the posterior body and the ramus of the mandible. The diagnosis of peripheral OKCs (other than intraosseous) are extremely rare and the current literature is limited. The most common location is the gingiva, but mucosal, epidermal, and even intramuscular sites have also been described. Currently 15 cases have been described. The origin and nature of peripheral OKC still remains controversial. The differential diagnosis includes gingival cyst, mucoceles and epidermoid cyst. Soft tissue OKCs have a lower rate of recurrences; 12,5% vs. 62% in intraosseus OKCs. We report a case of a 58-year-old woman with a peripheral OKC, located in the left masticatory space. We performed a review of the existing literature on peripheral odontogenic keratocysts.

** Key words:**Odontogenic keratocysts (OKCs), peripheral keratocyst, mandibular cyst.

## Introduction

Odontogenic keratocysts (OKCs) are benign cysts arising from the dental lamina and its remnants. They are most commonly located in the posterior body and the ramus of the mandible (1). OKCs are known for their difficult management, as they present rapid growth and a high rate of recurrence (16%-30%) (2).

The diagnosis of peripheral OKCs (other than intraosseous) is extremely rare and the current literature is limited. The most common location is the gingiva, but mucosal, epidermal and even intramuscular sites have also been described (3-5). The origin of these peripheral OKCs) is still a controversy.

The cases of OKC in the soft tissue other than the gingiva reported in the literature in the last 10 years are presented in  Table 1.

**Table 1:**
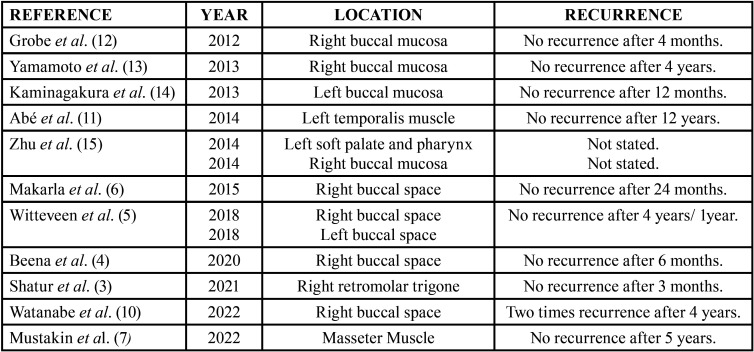
Cases of OKC in the soft tissue reported in the literature in the last 10 years.

In this article; we report the case of a 58 year old woman with a peripheral OKC, located in the left masticatory space, and a literature review on peripheral odontogenic keratocysts.

## Case Report

A 58-year-old woman was referred to our department due to a second recurrence of a mandibular keratocyst. She had undergone a first surgical in other Hospital in 2015, through an external cervical approach. In March 2022 she presented with a swelling in the left preauricular region. Given the suspicion of a new recurrence, a CT and an FNA were performed.

**Figure 1:**
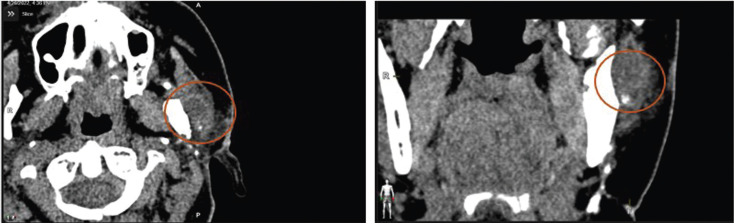
Preoperative CT scan. Red circle marks tumor lesion.

The CT scan revealed a loculated cystic lesion of 11x26x19 mm in contact with the lateral and posterior margin of the left mandibular ramus (Fig. 1). The FNA showed the presence of blood with no cell component, being insufficient for diagnosis. An MRI was performed, showing a mass with heterogeneous content in the left masticatory space, in contact with the mandibular ramus and with adhesions in the anterior margin of the left parotid gland.

**Figure 2:**
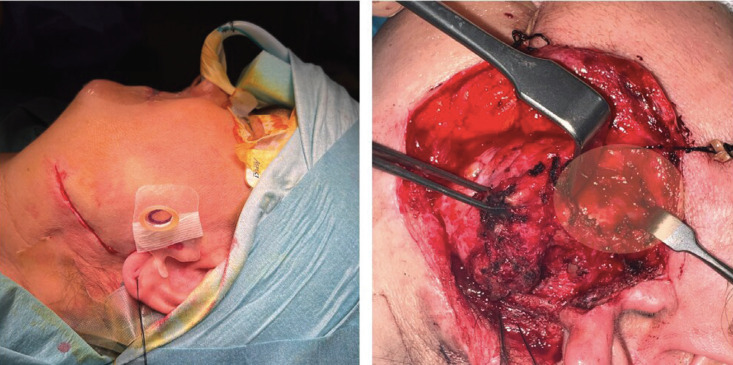
Left; surface marker guide. Right, intraoperative image of the cyst.

Surgery was decided to remove the lesion. A complete cystectomy was performed through the previous rhytidectomy approach. The cyst was found adherent to the left mandibular ramus and immediately deep to the trunk of the facial nerve and its buccal and zygomatic branches (Fig. 2).

**Figure 3:**
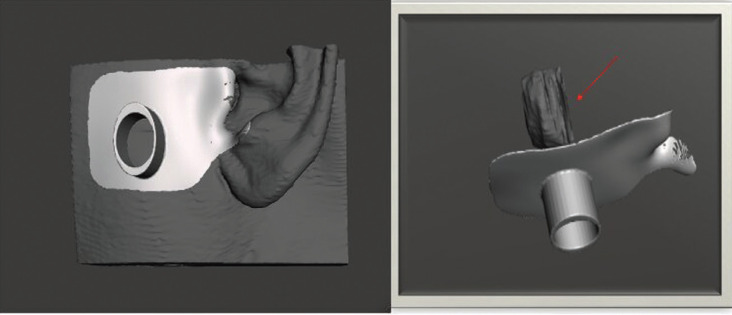
Surface marker. Red arrow marks the cyst.

Since the tumor was deeply located and not palpable, we designed a surface marker. The skin surface and the lesion were segmented using 3D Slicer. Then, a guide was designed which adapted over the skin surface and used the tragus as reference for adaption (Fig. 3). The guide owned a hole which marked the location of the mass. This guide was printed in biocompatible resin, cured and sterilized for its use in the operating room.

The specimen was routinely processed and stained with hematoxylin and eosin. Microscopically, the tissue sections showed typical histological features of keratocyst.

There was no recurrence during a follow-up for 3 months.

## Discussion

OKC is one of the most common odontogenic cysts. It has been redefined as a cyst in 2017 by the World Health Organization (WHO) from a neoplasm. The classification as a neoplasm in 2005 was due to their aggressive behavior, their high recurrence rate, and their associated mutations in the protein patched homolog 1 (PTCH) tumor suppressor gene. Such mutations in the PTCH gene have now been seen in non-neoplastic lesions, providing further evidence for this reclassification (3).

OKC arising from purely soft tissue other than gingival is extremely rare. The diagnosis of OKC in the extragnathic site is based on the typical histopathologic features identical to that of conventional OKC arising in the jaw. This histopathological diagnostic criteria for OKC were established un 1962 by Pindborg, Philipsen, and Henriksen (4).

The origin of peripheral OKCs is still under controversy. The different hypotheses are.

1) Remnants of the dental lamina may get displaced in the buccal mucosa and persist during embryogenesis. A recent embryological description of the developmental relationship between deciduous dentition and the oral vestibule may give an important clue. Accordingly, the remnants of the dental lamina entrapped in the buccal tissue during embryogenesis may develop a keratocyst with the features of OKC. Other odontogenic lesions such as ameloblastoma and odontoma have also been reported to develop in the buccal mucosa (5-8).

2) The epithelial lining of the OKC in the buccal space may originate from the sebaceous duct epithelium. Sebaceous glands are commonly located in the buccal mucosa as Fordyce spots

3) Keratocyst of epidermal origin may occur ectopically in the buccal mucosa, which is referred to as an epidermal inclusion cyst or a cutaneous keratocyst (8).

Regarding histology, these cysts have defined histopathological characteristics. The cystic lining consists of a parakeratinized stratified squamous epithelium of uniform thickness and a polarized basal layer and is devoid of of rete ridges. It is also not uncommon to find multiple invaginations of the epithelium. The lumen typically consists of a thick or straw-colored creamy fluid that may represent keratin (3,6).

Peripheral OKCs have a lower rate of recurrences; around 12,5% (5). This data differs from the recurrence rate recorded for intraosseous OKS (up to 62%) (1). This could be due to better resectability in the soft tissues or perhaps because we are talking about two entities with different biological behavior. Even so, more cases with long-term follow-up are required to obtain conclusions about the recurrence rate.

Some authors suggest that care should be taken to avoid contamination of surrounding tissue during OKC removal, as recurrences could be due to traumatic implantation of cystic tissue in the surrounding area (9).

When making the diagnosis of peripheral OKC, we must make a differential diagnosis with a series of similar lesions. Both gingival cysts and mucoceles arise in similar locations and have similar features; however, the histopathology differs as gingival cysts have thin, noninflamed walls lined by a thin squamous or cuboidal epithelium, and mucoceles have the diagnostic presence of salivary gland tissue and sialomucin (4,10).

Epidermoid cyst is another type of keratinised cysts with similar histopathological features; however, previous studies suggested that the differential diagnosis from epidermoid cysts could be done by their immunohistochemical profiles for CK17 and Ki-67 (13). OKC has a strong expression of CK17 and higher Ki-67-labelled cells (11).

Also orthokeratinized odontogenic cysts have similar presentations; however, the histopathology typically shows a thin, uniform epithelial lining with orthokeratinization and a subjacent granular cell layer (5,8).

In this article, a case of a peripheral OKC has been presented. The patient had suffered several recurrences and had been operated through an external cervical approach in the first surgery. When referred to our department, she presented with a mass in the left preauricular region. Keratocysts are lesions with a high rate of recurrence, as in this case, an even soft tissue recurrence must be suspected.

The use of a surface marker for soft tissue lesions can help locate deeply seated tumors, avoiding the need to use a navigator, which can sometimes lengthen surgery and requires the use of reference frames. Nonetheless, the guide does not offer depth information nor real-time tracking of the surgery.

In conclusion, we would like to highlight that since OKCs can occur in soft tissues as well, they should be included in the differential diagnosis of cystic lesions in the buccal space. The diagnosis of peripheral OKC can be established by proper radiological and histological evaluation.

The origin and nature of this disease as well as its recurrente rate are controversial, so further research is required.
